# Reduced-Dimensionality Quantum Dynamics Study of the ^3^Fe(CO)_4_ + H_2_ → ^1^FeH_2_(CO)_4_ Spin-inversion Reaction

**DOI:** 10.3390/molecules25040882

**Published:** 2020-02-17

**Authors:** Toshiyuki Takayanagi, Yuya Watabe, Takaaki Miyazaki

**Affiliations:** Department of Chemistry, Saitama University, Shimo-Okubo 255, Sakura-ku, Saitama 338-8570, Japan; y.watabe.827@ms.saitama-u.ac.jp (Y.W.); t.miyazaki.793@ms.saitama-u.ac.jp (T.M.)

**Keywords:** spin crossover, spin inversion, reaction dynamics, nonadiabatic transition, spin-orbit coupling, wave packet, crossing point, cumulative reaction probability

## Abstract

Many chemical reactions of transition metal compounds involve a change in spin state via spin inversion, which is induced by relativistic spin-orbit coupling. In this work, we theoretically study the efficiency of a typical spin-inversion reaction, ^3^Fe(CO)_4_ + H_2_ → ^1^FeH_2_(CO)_4_. Structural and vibrational information on the spin-inversion point, obtained through the spin-coupled Hamiltonian approach, is used to construct three degree-of-freedom potential energy surfaces and to obtain singlet-triplet spin-orbit couplings. Using the developed spin-diabatic potential energy surfaces in reduced dimensions, we perform quantum nonadiabatic transition state wave packet calculations to obtain the cumulative reaction probability. The calculated cumulative reaction probability is found to be significantly larger than that estimated from the one-dimensional surface-hopping probability. This indicates the importance of both multidimensional and nuclear quantum effects in spin inversion for polyatomic chemical reaction systems.

## 1. Introduction

Many chemical reactions can proceed through different spin multiplicity states during the course of a reaction, including electronically nonadiabatic transitions induced by spin-orbit coupling [1,2,3,4,5,6,7,8,9,10,11,12,13]. Since spin-orbit coupling is generally large in molecular systems that contain heavy elements, many catalytic reactions involving transition metal atoms occur on multiple potential energy surfaces with different spin multiplicities. Such a chemical reaction scheme has been historically called “two-state reactivity” or “multistate reactivity” [8,9,10,11,12,13]. It should also be mentioned that nonadiabatic spin-inversion transitions have alternatively been called intersystem crossing, spin crossover, or spin-forbidden transitions. The term “spin-forbidden” has long been used in chemistry because spin inversion cannot occur within a nonrelativistic Hamiltonian framework [1,2,3,5]. However, it should be emphasized that spin inversion is induced entirely by the large relativistic spin-orbit coupling of heavy elements. To fully understand the detailed mechanisms of catalytic reactions involving spin-inversion transitions, not only reaction pathways on a single potential energy surface with a specific spin state but also spin-inversion points between two or more potential energy surfaces with different spin states should be explored. In addition, the efficiency of the nonadiabatic spin-inversion transitions should be discussed quantitatively.

We have recently developed a practical computational approach for studying spin-inversion reaction mechanisms [14,15,16,17], where the lowest-lying eigenstate of the spin-coupled Hamiltonian matrix [18] is used in the identification of reaction pathways involving spin-inversion points. In this method, two potential energy surfaces with different spin states are coupled through a pre-assumed spin-orbit coupling parameter. With this method, the spin-inversion structure can be easily optimized as the transition state on the spin-coupled potential energy surface, which is a close approximation of the minimum energy crossing point (MECP) structure. In addition, we can calculate the intrinsic reaction coordinate (IRC) for the spin-inversion pathway, as in the case of a usual spin-conserved reaction. In the present study, we apply this method to a typical spin-inversion reaction, ^3^Fe(CO)_4_ + H_2_ → ^1^FeH_2_(CO)_4_, to perform nonadiabatic quantum dynamics calculations. This reaction has been investigated from a theoretical perspective [19,20,21,22,23,24,25]; however, most of the previous studies focused on static aspects, such as characterization of the potential energy surfaces around the crossing regions and spin-inversion efficiencies derived from a one-dimensional Landau–Zener-like approach. Recently, singlet-triplet spin-inversion rates for the very simple systems SiH_2_ and GeH_2_ have been calculated using the ab initio multiple spawning molecular dynamics method, which can approximately account for both electronically nonadiabatic transitions and nuclear quantum effects using the semiclassical frozen Gaussian wave packet method [26,27,28]. The calculated nonadiabatic spin-inversion rates were found to be more than twofold larger than those calculated by the one-dimensional Landau–Zener-like approach, suggesting the importance of multidimensional nonlocal transitions that cannot be described by a one-dimensional approach.

In this work, we discuss the importance of multidimensional nuclear quantum effects in the ^3^Fe(CO)_4_ + H_2_ → ^1^FeH_2_(CO)_4_ spin-inversion reaction by applying transition state wave packet calculations [29,30] combined with the above-mentioned spin-coupled Hamiltonian approach [14,15,16,17]. We here develop the reduced-dimensionality potential energy surfaces as a function of three nuclear degrees-of-freedom and then calculate the cumulative reaction probability, which is the most important physical quantity for calculating thermal rate constants [29,30]. The calculated cumulative reaction probability is compared with that derived from a one-dimensional approach.

## 2. Computational Procedure

The singlet and triplet potential energy surfaces for the H_2_ + Fe(CO)_4_ reaction were calculated by density functional theory (DFT) using the Gaussian 09 program [31]. Previous studies have already shown that the spin-inversion barrier height, which also corresponds to the MECP energy, is very sensitive to the calculated singlet-triplet splitting in the reactant Fe(CO)_4_ molecule [25]. Additionally, large-scale CCSD(T)/VTZ (for H, C, and O) calculations have shown that the singlet-triplet splitting is in the range 0.17–0.22 eV (depending on the quality of the Fe basis set). Thus, we searched for an appropriate DFT functional level that provides a reasonable singlet-triplet splitting value for Fe(CO)_4_. All benchmarking calculations were performed using the def2-TZVPP basis set, and we calculated the splitting values using the B97D, ωB97XD, OPBE, OLYP, M06, M06L, M06-2X, and TPSSh functionals. Among these functionals, we found that the M06/def2-TZVPP calculation yielded a value of 0.22 eV, which is in reasonable agreement with the CCSD(T) value. Therefore, the subsequent DFT calculations were performed at the M06/def2-TZVPP level. The singlet-triplet splitting values obtained from all the DFT functionals are presented in the Supplementary Material.

**Figure 1:**
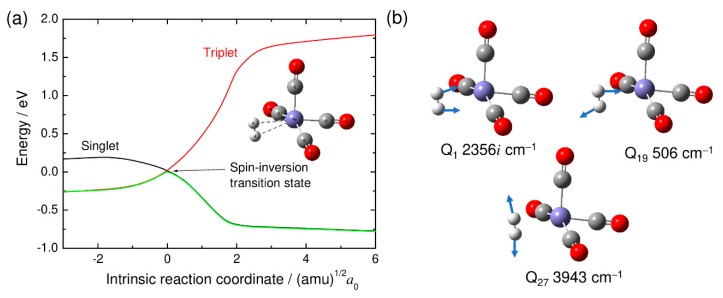
(**a**) M06/def2-TZVPP potential energy profiles for the ^3^Fe(CO)_4_ + H_2_ → ^1^FeH_2_(CO)_4_ reaction along the intrinsic reaction coordinate: triplet (red), singlet (black), and mixed-spin state (green). The spin-inversion (transition state) structure is also shown. (**b**) The three normal-mode vectors used to construct reduced-dimensionality potential energy surfaces.

The spin-coupled Hamiltonian approach [14,15,16,17] has been used to find the spin-inversion transition state structure, with the pre-assumed coupling parameter set to 100 cm^−1^. Figure 1 shows the optimized spin-inversion structure and the potential energy profiles of the spin-mixed, triplet, and singlet states along the IRC. The spin-inversion barrier height measured from the H_2_ + ^3^Fe(CO)_4_ reactant energy level was calculated to be 0.287 eV at the M06/def2-TZVPP level. Hereinafter, all energies are calculated relative to the spin-inversion energy level. The present reaction system has 11 atoms, and thus, 27 internal degrees of freedom. Needless to say, it is impossible to perform full-dimensional quantum dynamics calculations, so we have chosen three normal-mode coordinates obtained from vibrational frequency analysis at the spin-inversion point on the spin-coupled surface: *Q*_1_, *Q*_19_, and *Q*_27_. Note that all three of these normal modes are associated with hydrogen atom motions. More specifically, *Q*_1_ is the spin-inversion reaction coordinate giving an imaginary frequency and approximately corresponds to the H_2_ translational motion. *Q*_19_ and *Q*_27_ approximately correspond to the H_2_ rotational and vibrational motions, respectively.

The Hamiltonian of the three-dimensional normal-mode model employed in this work can be simply written as

(1) $$H=-\frac{\hbar^{2}}{2\mu }\left(\frac{\partial^{2}}{\partial s_{1}^{2}}+\frac{\partial^{2}}{\partial s_{2}^{2}}+\frac{\partial^{2}}{\partial s_{3}^{2}}\right)+V\left(s_{1},s_{2},s_{3}\right)$$

where ***V***(*s*_1_, *s*_2_, *s*_3_) is the potential energy surface matrix described by the spin-diabatic representation. *s*_i_ is the mass-weighted normal-mode coordinate for the index *i* (with *s*_1_ = *Q*_1_, *s*_2_ = *Q*_19_, and *s*_3_ = *Q*_27_), and μ is the reduced mass (= 1 atomic mass unit). The potential energy surface matrix for the singlet-triplet spin inversion case can be generally written by the following (4 × 4) matrix [7,32]:

(2) $$V\left(s\right)=\left(\begin{array}{cccc} V_{S}\left(s\right) & z_{1}\left(s\right) & iz_{2}\left(s\right) & z_{1}{}^{*}\left(s\right)\\ z_{1}{}^{*}\left(s\right) & V_{T}\left(s\right) & 0 & 0\\ -iz_{2}\left(s\right) & 0 & V_{T}\left(s\right) & 0\\ z_{1}\left(s\right) & 0 & 0 & V_{T}\left(s\right) \end{array}\right)$$

where the diagonal elements are the spin-free potential energies with *V_S_*(***s***) and *V_T_*(***s***) being the singlet and triplet surfaces, respectively. ***s*** collectively denotes the three normal-mode coordinates. Spin-orbit couplings *z*_1_ and *z*_2_ are respectively complex- and real-valued functions of the nuclear coordinates. As will be shown later, the absolute values of *z*_2_ and the real part of *z*_1_ are found to be very small compared with the imaginary part of *z*_1_ over the nuclear coordinates examined here. In addition, the imaginary part of *z*_1_ is only weakly dependent of the nuclear coordinates. These findings naturally lead to the following (2 × 2) potential energy matrix:

(3) $$V\left(s\right)=\left(\begin{array}{cc} V_{S}\left(s\right) & i\sqrt{2}Im\left(z_{1}\right)\\ -i\sqrt{2}Im\left(z_{1}\right) & V_{T}\left(s\right) \end{array}\right)$$

where *z*_1_ is taken to be a constant value. Thus, the potential energy matrix in the Hamiltonian of Equation (1) can be written as Equation (3).

The cumulative reaction probability, which is the sum of the probabilities of the transitions from all the reactant states to all the product states, can be calculated using various time-dependent and time-independent quantum dynamics techniques [33,34]. In this work, we employ the time-dependent transition state wave packet approach [29,30], because we need to propagate wave packets in only the limited region of the potential energy surfaces around the transition state. The initial wave packets are constructed as direct products of the two-dimensional Hamiltonian eigenstates at a specific value of *s*_1_ and the flux operator eigenstate with a positive eigenvalue. After constructing the initial wave packets, we propagate them both forward and backward in time. The cumulative reaction probability *N*(*E*) can be easily calculated as

(4) $$N\left(E\right)=\underset{i}{\sum }N_{i}\left(E\right)=\underset{i}{\sum }\langle \psi_{i}\left(E\right)\left|F\right|\psi_{i}\left(E\right)\rangle$$

where *ψ_i_* is the energy-dependent wave function for the *i*-th initial vibrational state, which can be easily obtained from the Fourier transform of the time-dependent wave packet. The flux operator *F* is defined as

(5) $$F=\frac{1}{2\mu }\left[\delta \left(s_{1}-s_{1}^{0}\right)p_{s_{1}}-p_{s_{1}}\delta \left(s_{1}-s_{1}^{0}\right)\right]$$

where *p*_s1_ is the momentum operator conjugate to the coordinate *s*_1_. In this work, the dividing surface is located at *s*_1_^0^ = 0. In References [29] and [30], the wave packets are propagated on a single adiabatic potential energy surface; however, here the wave packet is defined in the diabatic representation and, therefore, has two components on the singlet and triplet diabatic potential energy surfaces.

**Figure 2:**
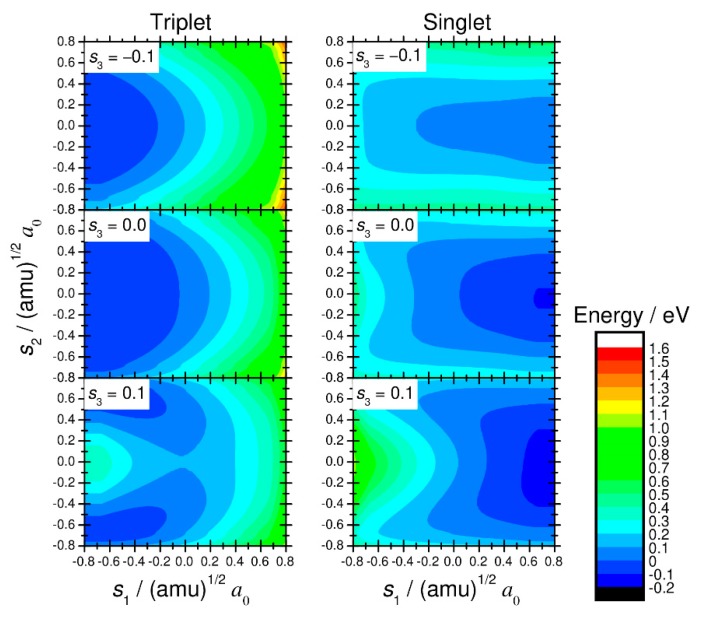
Two-dimensional contour plots of the triplet and singlet potential energy surfaces for the ^3^Fe(CO)_4_ + H_2_ → ^1^FeH_2_(CO)_4_ reaction calculated by density functional theory at the M06/def2-TZVPP level.

The M06/def2-TZVPP-level DFT calculations were done at the geometrical grid points described by the three normal-mode coordinates, where 11 points were used for each of three coordinates: *s*_1_, *s*_2_, and *s*_3_. The calculations were done in the range [−0.8, 0.8] (amu)^1/2^*a*_0_ for *s*_1_ and *s*_2_ and in the range [−0.4, 0.4] (amu)^1/2^*a*_0_ for *s*_3_. The cubic spline interpolation technique was used to obtain the potential energy values at a desired set of nuclear coordinates. The obtained potential energy surfaces for the singlet and triplet spin states are presented in Figure 2.

**Figure 3:**
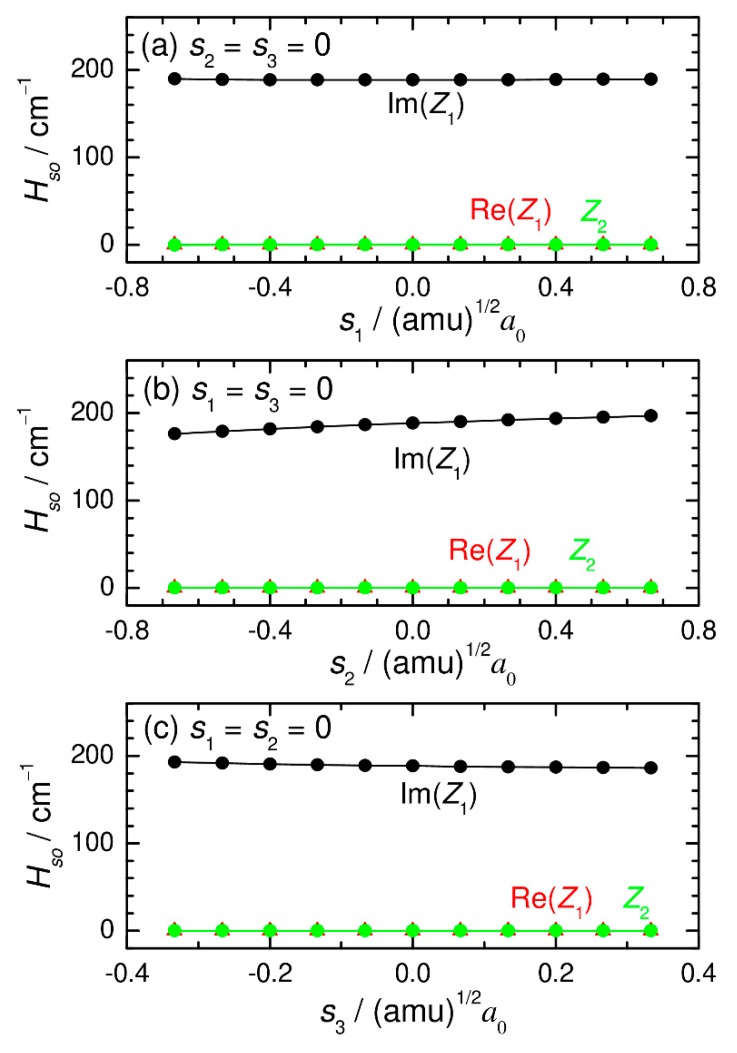
Spin-orbit couplings (*z*_1_ and *z*_2_; see main text) plotted as a function of normal-mode coordinates. The coupling values were calculated at the CASSCF(10_e_,8_o_)/def2-TZVPP level.

**Figure 4:**
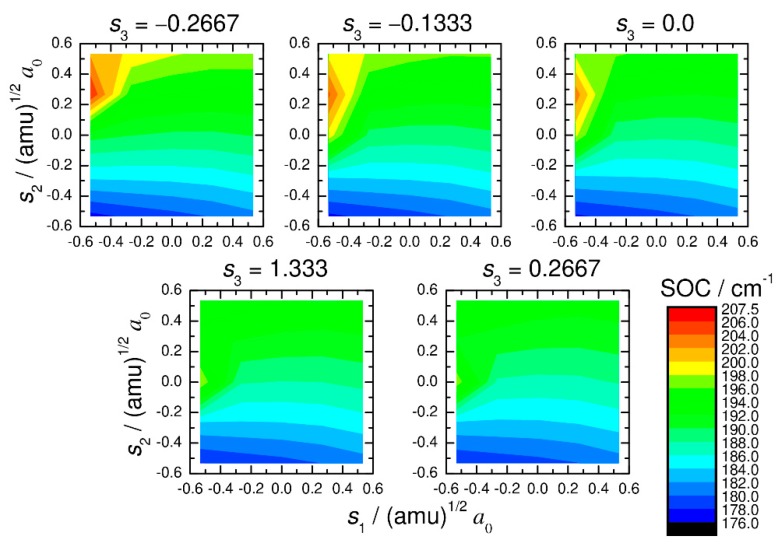
The largest component of the spin-orbit coupling matrix elements (imaginary part of *z*_1_; see main text) plotted as a function of normal-mode coordinates. The spin-orbit coupling values were calculated at the CASSCF(10_e_,8_o_)/def2-TZVPP level.

The spin-orbit couplings were determined within the Breit–Pauli approximation using the state-interacting method at the state-averaged CASSCF/def2-TZVPP level of theory. These electronic structure calculations were performed using the MOLPRO program [35]. Two states (one singlet and one triplet) were equally averaged, where 10 active electrons were distributed among 8 active orbitals in the CASSCF wavefunction. These active orbitals include 3d and 4s orbitals of Fe and two 1s orbitals of H. The calculated results are presented in Figure 3 and Figure 4. We can see that only the imaginary part of *z*_1_ (see Equation (2)) is important and that the real part of *z*_1_ and the absolute value of *z*_2_ are always very small and can be safely ignored in the quantum dynamics calculations, as mentioned above. In addition, the imaginary part of *z*_1_ is found to depend only weakly on the nuclear coordinate. Therefore, we carried out all the quantum dynamics calculations using a constant value (188.4 cm^−1^) at the coordinate origin (*s*_1_ = *s*_2_ = *s*_3_ = 0).

The wave packets were described using grid-based discrete-variable representations (DVRs) [33,34,36], where the standard particle-in-a-box basis sets were used for all three normal-mode coordinates. After the initial wave packet was prepared, it was propagated using the extended split-operator method [37,38] through the simple extension of the standard split-operator method on a single surface [33,34] to the multiple potential energy surface problem. A quadratic polynomial was used as a negative imaginary absorbing potential [39] to avoid artificial reflection of the wave packet at the edge of the potential energy surfaces. The final DVR parameters used were 256, 64, and 64 grid points for the *s*_1_, *s*_2_, and *s*_3_ coordinates, respectively. The total step for time propagation was 60,000 with Δ*t* = ± 0.5 atomic time units.

## 3. Numerical Results

**Figure 5:**
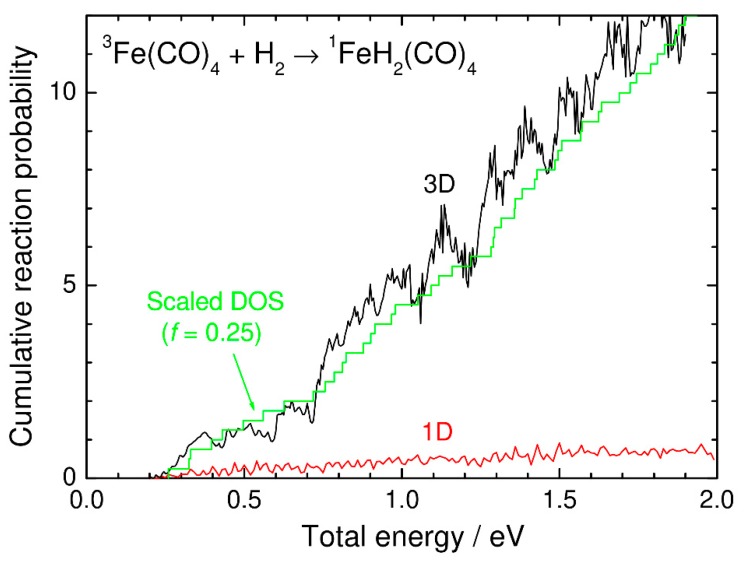
Comparison of the cumulative reaction probabilities as a function of energy: three-dimensional quantum transition state wave packet results (black), one-dimensional results (red), and scaled density of states (DOS) (green).

Figure 5 shows the cumulative reaction probability as a function of energy calculated using the present reduced-dimensionality quantum dynamics model. The 41 lowest initial states were summed to obtain numerically converged results in this energy range (see Equation (4)). Showing the expected behavior, the calculated cumulative reaction probability increases with increasing energy. We also note an oscillating feature indicating that the reaction dynamics are dominated in part by quantum mechanical resonances, which correspond to the quasi-bound states localized around the transition-state region. This is not surprising, because the upper adiabatic potential energy surface produced from the coupling between the singlet and triplet surfaces has a bound potential well (see Figure 1).

**Figure 6:**
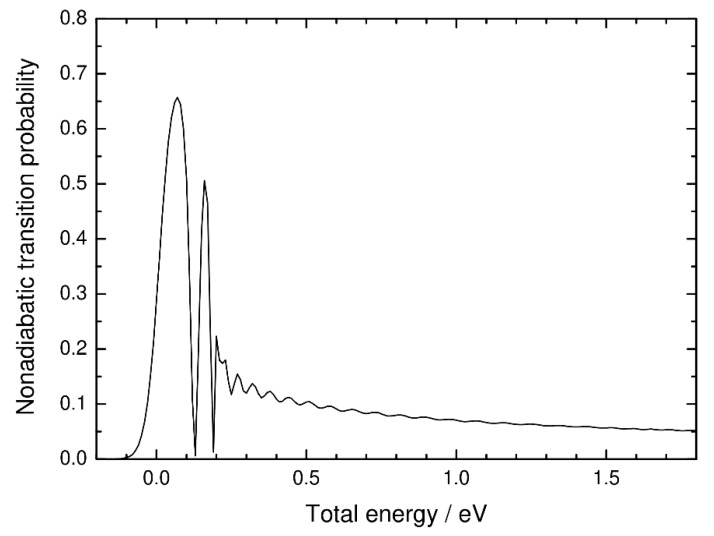
Nonadiabatic transition probability as a function of energy calculated with the time-independent *R*-matrix propagation method using the one-dimensional spin-diabatic potential energy curves shown in Figure 1.

In Figure 5, we also plot the cumulative reaction probability calculated from the one-dimensional nonadiabatic transition (surface-hopping) probability as

(6) $$N^{1D}\left(E\right)=\underset{i}{\sum }P^{1D}\left(E-\varepsilon_{i}\right)$$

where *P*^1D^ is the nonadiabatic transition probability calculated from the one-dimensional singlet and triplet potential energy curves along the IRC (see Figure 1), and *ε_i_* is the *i*-th vibrational energy level at *s*_1_ = 0. The transition probability was calculated using the time-independent *R*-matrix propagation method [34] with a constant spin-orbit coupling and is shown in Figure 6 as a function of energy. The results in Figure 6 show an expected feature of the Landau–Zener approach where the transition probability is large around the crossing energy level [3], although resonance behavior (and interference at high energies) can be also seen, as in the three-dimensional case. However, as shown in Figure 5, the most important point is that the cumulative reaction probability obtained from the one-dimensional model is significantly smaller than that obtained from our three-dimensional model. This result is essentially similar to the results of the previous ab initio multiple spawning calculations for GeH_2_ and SiH_2_ [26,27,28] mentioned in the Introduction.

In Figure 5, the scaled density of states on the *s*_1_ = 0 surfaces is compared with the cumulative reaction probabilities calculated from the three-dimensional and one-dimensional models. The scaled density of states can be defined as

(7) $$N_{s}^{‡}\left(E\right)=\underset{i}{\sum }fH\left(E-\varepsilon_{i}\right)$$

where *f* is an appropriate scaling factor and *H* is the Heaviside step function. If *f* is taken to be unity, then *N*_s_^‡^(*E*) exactly corresponds to the number of open states at the dividing surface. We found that the scaled density-of-states function with *f* = 0.25 approximately agrees with the cumulative reaction probability curve obtained from our three-dimensional quantum dynamics approach. This indicates that the average nonadiabatic transition probability can be assumed to be a constant value of about 0.25 independent of energy. The simple 1D Landau–Zener approach, on the other hand, should be used with caution when applying the nonadiabatic transition state theory [3]. As shown in Figure 6, the one-dimensional nonadiabatic transition probability is small both at energies below the spin-inversion barrier and at higher energy due to the well-known double-passage behavior through the crossing region, where the overall reaction probability is the product of *P* and 1-*P* (*P* is the nonadiabatic transition probability). The qualitative agreement between the three-dimensional quantum dynamics result and the scaled density of states suggests that double-passage events are overcounted in the one-dimensional model. This also indicates that coupling between the nonadiabatic spin-inversion motion and other nuclear motions plays an essential role leading to multidimensional nonlocal transitions. The findings of our quantum dynamics calculations are thus in line with the previous ab initio multiple spawning studies on the spin-inversion dynamics of the GeH_2_ and SiH_2_ systems [26,27,28].

## 4. Conclusions

In this work, we have theoretically studied the typical spin-inversion reaction ^3^Fe(CO)_4_ + H_2_ → ^1^FeH_2_(CO)_4_. The spin-inversion point, which approximately corresponds to the MECP between the singlet and triplet potential energy surfaces, was successfully optimized as a transition state by using the spin-coupled Hamiltonian approach. This approach thus provides the effective normal-mode vibrational frequencies at the spin-inversion point. Using the vibrational frequency information, we constructed spin-diabatic potential energy surfaces by taking three nuclear degrees-of-freedom as active coordinates. Then, we calculated the reduced-dimensionality cumulative reaction probability using the quantum transition state wave packet approach. We found that the cumulative reaction probability calculated in this way is significantly larger than that estimated from the one-dimensional surface-hopping probability. The present study thus suggests the importance of both multidimensional and nuclear quantum effects in polyatomic chemical reactions involving changes in spin states.

## Supplementary Materials

The following is available online. Table S1: Comparison of the singlet-triplet energy gap (Δ*E* in kcal/mol) in the Fe(CO)_4_ reactant obtained from various DFT functionals (without zero-point energy correction).
