# Supplementary material for: Reduced-Dimensionality Quantum Dynamics Study of the 3Fe(CO)4 + H2 → 1FeH2(CO)4 Spin-inversion Reaction
Source: Molecules. 2020 Feb 17;25(4):882. doi: 10.3390/molecules25040882 (PMC7070764; doi:10.3390/molecules25040882)
Supplement: Supplementary file 1 [file molecules-25-00882-s001.pdf]

Reduced-dimensionality quantum dynamics study of the $^3Fe(CO)_4 + H_2 \rightarrow {}^1FeH_2(CO)_4$ spin-inversion reaction

Toshiyuki Takayanagi [1,*], Yuya Watabe [1] and Takaaki Miyazaki [1]

[1] Department of Chemistry, Saitama University, Shimo-Okubo 255, Sakura-ku, Saitama City, Saitama 338-8570, Japan

* Correspondence: tako@mail.saitama-u.ac.jp; Tel.: +81-48-858-9113

Supplementary Material

Table S1. Comparison of the singlet-triplet energy gap (Δ*E* in kcal/mol) in the $Fe(CO)_4$ reactant obtained from various DFT functionals (without zero-point energy correction).

DFT functional	Δ*E* (kcal/mol)
TPSSh / def2-TZVPP	7.18
M06-2X / def2-TZVPP	24.24
M06L / def2-TZVPP	8.30
M06 / def2-TZVPP	5.14
OPBE / def2-TZVPP	–0.095
OLYP / def2-TZVPP	0.74
B97D / def2-TZVPP	0.83
ωB97XD / def2-TZVPP	8.53
CCSD(T)	4.02–5.03[a]

[a] Taken from the paper by Carreón-Macedo and Harvey (*Phys. Chem. Chem. Phys*. **2006**, *8*, 93–100).
